# Discrepancies between parent and child report of anxiety in children with high functioning autism spectrum disorder

**DOI:** 10.1192/j.eurpsy.2021.573

**Published:** 2021-08-13

**Authors:** R. Ayoub, T. Brahim, H. Ben Abid, S. Bousleh, A. Guedria

**Affiliations:** Child And Adolescent Psychiatry Department, Fattouma Bourguiba University Hospital, Monastir, Tunisia

**Keywords:** Anxiety, High Functioning Autism Spectrum Disorder, ASC-ASD

## Abstract

**Introduction:**

Many children with autism spectrum disorder (ASD) experience high levels of anxiety. However, inconsistencies between parent and child reports may complicate the assessment of anxiety in this population.

**Objectives:**

The aim of this study is to investigate discrepancies between parent and self-reported anxiety among children with high functioning ASD.

**Methods:**

Children aged between 8 and16 years with high functioning ASD, followed in the outpatient unite of child psychiatry at the University Hospital of Monastir, Tunisia and their parents, were invited to complete the Arabic version of the Anxiety Scale for Children with Autism Spectrum Disorder (ASC-ASD). The ASC-ASD is a 24-item questionnaire with six sub-scales, designed specifically for the assessment of anxiety symptomatology in children with ASD. A total score of ≥ 20 indicate significant levels of anxiety.

**Results:**

We recruited 66 children and their parents. The mean age was 10 years old. High rates of anxiety were found: children’s reports revealed that 70% of them present anxiety and 60 % were coated anxious by parent’s reports. Performance Anxiety, Uncertainty and Social Anxiety were the most frequent types of anxiety reported by both parents and children. We did not find a significant difference between the parents and children rating of anxiety except for the apprehension subscale.

**Conclusions:**

This is one of the first studies to compare between parent and self-reported anxiety among children with ASD using an autism-specific measure of anxiety. No discrepancies have been found between parents and children report, however others studies are needed to investigate discrepancies in children with severe ASD.

